# The distribution of hepatitis C virus infection in Shanghai, China: a time-spatial study

**DOI:** 10.1186/s12879-021-06577-8

**Published:** 2021-09-18

**Authors:** Ling-Xiao Qu, Yang Shi, Kai-Yun Chen, Yi-Han Lu, Hong Ren

**Affiliations:** 1 Department of Viral Hepatitis Control and prevention, Division of TB and HIV/AIDS Prevention, Shanghai Municipal Center for Disease Control and Prevention, Shanghai, China; 2 Department of Epidemiology, Fudan University School of Public Health, Shanghai, China; 3 Ministry of Education Key Laboratory of Public Health Safety (Fudan University), Shanghai, China

**Keywords:** Hepatitis C Infection, Cluster, Time-spatial distribution, Genotype

## Abstract

**Background:**

Shanghai, as a pilot city of China to achieve the goal of eliminating hepatitis C, its strategy of allocating medical resources is a pressing problem to be solved. This study aims to infer the time-spatial clustering patterns of HCV-infected cases, and grasp the dynamic genotype distribution of HCV, thereby inform elimination strategies of HCV with efficacy and efficiency.

**Methods:**

Reported HCV cases including their demographic information in Shanghai city from 2005 to 2018 were released from the National Infectious Disease Reporting Information System, population data at community scale, geographical layers of hospitals, communities and districts were gathered from former research. Blood samples of HCV-infected individuals were collected during 2014–2018 from 24 sentinel hospitals, HCV-antibody test, qualitative nucleic acid test and NS5B/5’UTR gene amplification were performed accordingly to determine the genotypes of the specimen. Furthermore, global and local spatial self-correlation analysis of both acute and chronic HCV infections were conducted at community scale year by year, then time-spatial clusters of acute and chronic HCV infections and HCV genotype distribution of specimen collected from sentinel hospitals by districts were mapped by using Arcmap10.1.

**Results:**

A total of 2631 acute HCV cases and 15,063 chronic HCV cases were reported in Shanghai from 2005 to 2018, with a peak in 2010 and 2017, respectively. The mean age of chronic HCV patients was 49.70 ± 14.55 years, 3.34 ± 0.32 years older than the acute (*t* = 10.55, *P*-value < 0.01). The spatial distribution of acute HCV infection formed one primary cluster (Relative Risk = 2.71), and the chronic formed one primary cluster and three secondary clusters with Relative Risk ranged from 1.94 to 14.42, meanwhile, an overlap of 34 communities between acute and chronic HCV clusters were found with time period spans varied from 6 to 12 years. Genotype 1 (N = 257, 49.71%) was the most prevalent HCV genotype in Shanghai, genotype 3 infections have increased in recent years. Baoshan district presented cluster of acute HCV and the highest proportion of genotype 2, Pudong new area was the cluster of chronic HCV and occupied the highest proportion of genotype 3.

**Conclusions:**

Despite the low prevalence of HCV infection, it is still needed to push forward the elimination process in Shanghai, as there is a certain amount of HCV infected people waiting to be treated. The time-spatial clustering patterns and the dynamic of HCV genotype distribution together indicated a changing constitution of different transmission routes of HCV infection, thus, a focused strategy may be needed for high-risk population related to genotype 3 infection like drug users, in addition to an enforcement of the existing measures of preventing the iatrogenic and hematogenic transmission of HCV.

## Background

Hepatitis C Virus (HCV) infection can cause both acute and chronic hepatitis, and is constantly one of the major causes of liver cirrhosis, hepatocellular carcinoma (HCC) and liver-related death [1, 2], representing an increasing health burden. More than 71 million people were infected with HCV chronically as estimated [3]. In 2016, the World Health Assembly approved the Global Health Sector Strategy to eliminate hepatitis infection (including HCV infection) as a major public health threat by 2030, which requires “a 90% reduction in new cases of chronic hepatitis C, a 65% reduction in hepatitis C deaths, and treatment of 80% of eligible people with chronic hepatitis C infections” [4]. Accordingly, strategies were recommended by World Health Organization (WHO) for different countries and regions based on differentiated scenarios of HCV endemicity [5]. Direct-acting antivirals (DAAs) therapy was first approved by U.S. Food and Drug Administration (FDA) in 2011 [6], since then, pangenotypic DAAs were approved successively and the prices were reduced substantially through these years, which enabled the planning and rolling out strategies of expanding test and treat for HCV infection.

China had made series efforts to approach the elimination target. Since 1993, measures had been implemented to cut off the HCV transmission routes of iatrogenic and hematogenic, including the implementation of mandatory HCV screening for blood transfusion, test routine before operations, as well as other precautions to prevent blood-borne disease [7]. As a result, the positive rate of HCV antibody (anti-HCV) in general population aged between 18 and 60 had drastically felt from intermediate seroprevalence (3.20%) in 1992 to low seroprevalence (0.43%) in 2006 [8, 9], most provinces of China have become low-prevalence areas (as indicated by WHO [5], settings with a lower than 2% seroprevalence of anti-HCV in general population) for HCV infection except for a few with relative higher prevalence due to former blood safety issues [10]. The first DAAs became available in 2015, and in 2019, three DAA combinations were introduced to the National Medical Insurance catalogue through government negotiation, with a price reduction of over US$ 7650 (over 85%) [11], greatly improved the accessibility and affordability of effective treatment. A new National Plan for the Prevention and Control of Viral Hepatitis from 2017 to 2020 were developed [12], the treatment guidelines were updated in 2019 [13], and an elimination plan specific to HCV for ten years will be established.

Shanghai is one of the four municipalities directly under the central government of China, located in the east of China, as a center of the international economy, finance, trade, and technology. According to the monitoring reports based on the surveillance system for viral hepatitis and Acquired Immune Deficiency Syndrome (AIDS) that established by Shanghai Municipal Center for Disease Control & Prevention (SCDC), in 2019, the seroprevalence of anti-HCV was 0.16% for the community-based general population, corresponding to nearly 40,000 HCV-infected individuals who live in Shanghai, the seroprevalence of anti-HCV was 2.22% (1459/65,660) in the high-risk population and drug users possessed the highest seroprevalence of 18.81% (1008/5358) among them.

HCV displays a high genetic diversity due to the high mutation rate of the viral polymerase and the high turnover of the virus. Viral variants are classified into seven genotypes (named1–7) and then further into at least 67 subtypes, (labelled as a, b, c, etc.) [14]. Worldwide, the distributions of HCV genotype show different geographic patterns. GT1, GT2, GT3 have a broad global distribution, whereas GT4, GT5 and GT6 are seen only in certain areas. Based on former studies, GT5 is mainly found in the Middle East and North Africa, GT5 is seemingly confined to South Africa, while GT6 is mainly distributed in southern China and South-East Asia. To date, only four HCV genotypes (1, 2, 3 and 6) have been reported in China, among which subtypes 1b, 2a, 3b, 6a and 3a are the most prevalent subtypes. Previous studies have indicated that the most prevalent HCV subtype was 1b, followed by 2a [15], while in the past few years, studies have shown new trends of HCV infection in some regions of China [16]. And many studies found association between genotype distribution and transmission route within certain region [17].

In addition to the identified HCV-infected cases, a majority of HCV-infected people are unaware [18] of their real conditions, it is difficult to find out or trace the asymptomatic infected individuals by using traditional methods, especially in the low-prevalence areas, and massive transient populations from both inside and outside of the Shanghai city complicated the situation. Recently, the geospatial-analysis has been successfully practiced in identifying the epidemiological patterns of both acute and chronic communicable diseases, such as H7N9 [19], viral hepatitis E [20], and tuberculosis [21]. Corresponding to the establishment of national plan for elimination HCV, Shanghai, as a pilot city of China to achieve the goal of eliminating hepatitis C, its strategy of allocating medical resources is a pressing problem to be solved. Identifying HCV time-spatial distribution could inform the allocation of related medical resources. And it is considered important to elucidate the changing scenario of different HCV genotypes and subtypes as the knowledge may indicate the major route of HCV transmission and inform the specific screening and treatment strategies.

This study aims to infer the time-spatial clustering patterns of HCV-infected cases, and grasp the dynamic genotype distribution of HCV, thereby inform elimination strategies of HCV with efficacy and efficiency.

## Methods

### Surveillance system of hepatitis C

Hepatitis C is a National Notifiable Infectious Disease Class B in China, physicians must report each clinically-diagnosed and laboratory-confirmed patient’s information through the web-based National Infectious Disease Reporting Information System (NIDRIS) to the Chinese Center for Disease Control and Prevention (CCDC) within 24 h. Community health care providers then conduct an epidemiological investigation and health education.

Acute and chronic HCV infection are defined by the diagnostic criteria and principles for the management of hepatitis C, acute HCV infection is diagnosed as a new HCV infection with suspected exposure history in the last 6 months or biopsy results of acute hepatitis, and chronic HCV infection is defined as continued HCV infection with suspected exposure history over 6 months after HCV infection. From 2005 to 2018, there were a total of 86 hospitals in Shanghai city have provided hepatitis C diagnosis and treatment services.

In 2014, a new surveillance system for viral hepatitis and AIDS was established based on the national surveillance system by SCDC, which was used to obtain the demographic information, identify risk factors and early detect the HCV-infected individuals. Since then, 24 sentinel hospitals were selected to undertake the monitoring tasks. They were all secondary (n = 9) or tertiary (n = 15) hospitals located in all the 16 administrative districts in Shanghai, in which the number of diagnosed patients covered more than 70% of all the newly reported HCV cases in Shanghai. Each sentinel hospital must report the information of the inpatients including case number as well as serological examination results to SCDC monthly and facilitate collecting blood samples (5–10 specimen per hospital per year) of newly diagnosed cases (including both acute and chronic hepatitis C) to SCDC as required.

### HCV genotype determination

First, all the blood samples were examined by Diagnostic Kit for HCV-antibody (ELISA, HCV antibody: BEIJING WANTAI DRD CO., LTD.). Second, the samples tested positive for HCV-antibody (S/cutoff value ≥ 1.0), a qualitative nucleic acid test by fluorescence PCR was performed using HCV nucleic acid testing kit (Shanghai Zhijiang Biotechnology Co., Ltd.). Third, viraemic samples were genotyped using Hepatitis C virus NS5B and 5'UTR gene amplification kits (Shanghai Berger Medical Technology Co., Ltd.). If the two genotyping results were consistent, the genotype was determined accordingly, if not, the genotype was determined with the 5’UTR-based genotyping. In addition, the genotype was determined on only one genotyping result due to insufficient volume for examination.

### Data collection, preparation and analyses

First, cases of reported HCV during 2005–2018 including their demographic information (such as sex, age, date of onset, reporting hospital, ZIP code of residential address, etc.) were released from the NIDRIS. The numbers of population from year 2005 to 2018 were provided by the Bureau of Statistics of Shanghai. The characteristics of HCV infected cases were described to supply a general profile of HCV infection in Shanghai.

Second, we allocated cases with ZIP code belonging to Shanghai to certain community by years. Rate of HCV infection per 100,000 population at the community level were calculated by using population denominators at community scale. And geographic layers of 86 HCV hospitals (including 24 sentinel hospitals), 16 districts, and 240 communities were obtained from our prior research [20] conducted by SCDC. Data was prepared by joining Geographic layers and rates of HCV infection for further spatial analyses.

Then we used tests of spatial auto-correlation and hot-spot analyses using inverse distance-based space method, to identify the geolocation of statistically significant hotspot clusters of HCV cases and case reporting hospitals. Additionally, we conducted spatiotemporal clustering patterns at community scale across 2005–2018 by using the log-likelihood ratio (*LLR*) test based on a discrete *Poisson* model to analyze spatial aggregation of the reported cases of HCV.

Furthermore, HCV-infected blood samples collected during 2014–2018 were genotyped and subtyped, subtype results were analyzed by years to describe the dynamics of HCV distribution of different subtypes, while the results of HCV genotype were allocated according to the patients’ ZIP code of the reporting hospitals and mapped to demonstrate the HCV genotype distribution.

The data was established and cleaned using Microsoft Excel 2016, descriptive statistical analysis including the calculation of the incidence and prevalence of acute and chronic HCV were determined by SAS 9.3 software (SAS Institute Inc., USA). The establishment of geographic layer, spatial auto-correlation and hot-spot analyses were conducted by ArcMap 10.1(ESRI, USA). Time-spatial clustering analysis was performed by SaTScan 9.4 (https: //www.satscan.com.org/). Time-spatial clusters of acute and chronic HCV infections, and HCV genotype distribution of specimen collected from sentinel hospitals by districts were mapped by using Arcmap10.1.

## Results

### General patterns of hepatitis C infection

A total of 2631 acute HCV cases were reported from 2005 to 2018, and 15,063 chronic HCV cases were reported from 2013 to 2018 in Shanghai, both including permanent residents and mobile population. Among them, males accounted for 59.45% (1564/2631) of the acute patients and 61.05% (9196/15,063) of the chronic ones. The mean age of chronic HCV patients was 49.70 ± 14.55 years, which was 3.34 ± 0.32 years older than the acute (*t* = 10.55, *P*-value < 0.01). The incidence of acute HCV cases fluctuated between 2.16 to 0.12 per 100,000 persons with a peak in 2010. The prevalence of chronic was from 7.52 to 12.45 per 100,000 persons, showed an upward trend with a peak in 2017, and the detailed information was shown in Table 1.

**Table 1:** Characteristics of cases infected with Hepatitis C Virus, Shanghai, China, 2005–2018

Year	Acute cases				Chronic cases				Population denominator
	Male	Female	Age (mean ± sd)	Incidence (/100,000)	Male	Female	Age (mean ± sd)	Prevalence (/100,000)	
2005	129	51	46.91 ± 16.78	180 (1.05)	–	–	–	–	17,100,766
2006	170	91	43.87 ± 18.04	261 (1.47)	–	–	–	–	17,780,009
2007	162	127	44.44 ± 17.62	289 (1.56)	–	–	–	–	18,580,800
2008	225	142	46.29 ± 17.57	367 (1.98)	–	–	–	–	18,579,999
2009	207	153	46.81 ± 17.18	360 (1.91)	–	–	–	–	18,884,597
2010	249	165	47.05 ± 16.82	414 (2.16)	–	–	–	–	19,210,015
2011	105	67	46.10 ± 17.01	172 (0.75)	–	–	–	–	23,019,196
2012	95	89	46.72 ± 18.51	184 (0.77)	–	–	–	–	23,804,303
2013	72	65	46.67 ± 17.25	137 (0.57)	1069	747	47.41 ± 14.84	1816 (7.52)	24,151,500
2014	33	28	46.10 ± 16.06	61 (0.25)	1268	903	48.63 ± 14.60	2171 (8.95)	24,256,797
2015	35	19	51.76 ± 13.18	54 (0.22)	1389	915	48.63 ± 14.45	2304 (9.54)	24,152,700
2016	28	28	50.55 ± 16.33	56 (0.23)	1786	1224	49.95 ± 14.60	3010 (12.44)	24,197,000
2017	41	25	48.32 ± 15.65	66 (0.27)	1877	1134	50.90 ± 14.46	3011 (12.45)	24,183,297
2018	13	17	45.67 ± 16.04	30 (0.12)	1807	944	51.34 ± 14.10	2751 (11.35)	24,239,994
total	1564	1067	46.36 ± 17.24	2631	9196	5867	49.70 ± 14.55	15063	–

### The time-spatial pattern of hepatitis C infection

Global spatial auto-correlation analysis showed that both acute and chronic HCV infections at community scale were clustering (positively correlated) in most of the years. Moran's *I* index for acute HCV infections varied from 0.008 to 0.074, and were significant (*P*-value < 0.05) for all individual years between 2007 and 2013, as well as in 2017. Moran's *I* index for chronic HCV infections varied from 0.032 to 0.052, the value were significant for year 2013 to 2016.

**Fig. 1:**
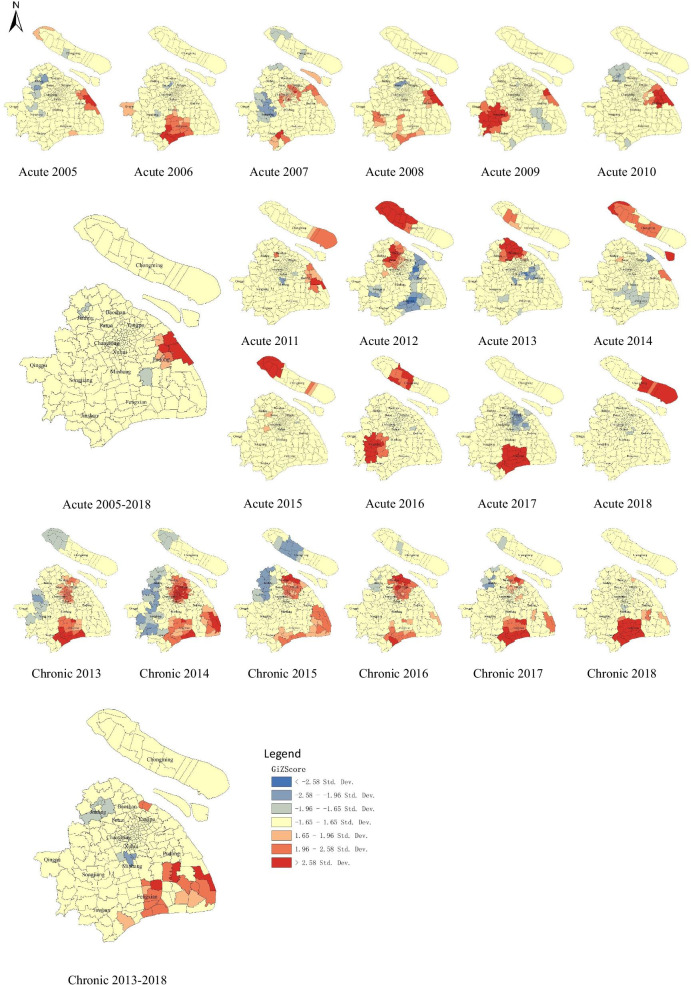
Local spatial auto-correlation analysis of HCV infection distribution Shanghai, China, 2005–2018

Local spatial auto-correlation analysis (Fig. 1) identified statistically significant (*P*-value < 0.05) persistent hot spots of acute HCV in east part of Shanghai from 2005 to 2011, and significant persistent hot spots of chronic HCV in south part and northeast part of Shanghai from 2013 to 2018.

**Fig. 2:**
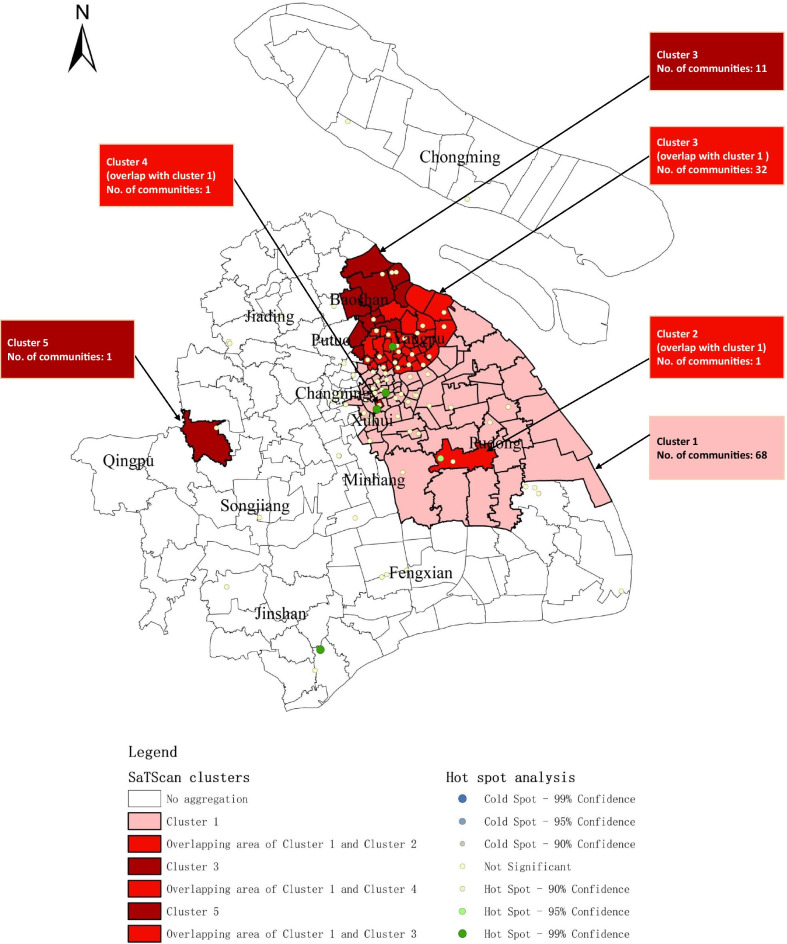
HCV infection spatiotemporal clusters with high rates identified by SaTScan discrete *Poisson* method, Shanghai, China, 2005–2018. i) No. communities: number of communities within cluster; ii) Cluster 1 belongs to primary clusters of acute HCV infection; iii) Cluster 2 belongs to primary clusters of chronic HCV infection, and cluster 3, 4 and 5 belong to secondary clusters of chronic HCV infection; iv) Hospital A, B, C, D and E are arranged from top to bottom of the figure

Time-spatial clustering analysis (Fig. 2, Table 2) showed that the spatial distribution of acute HCV infections formed one primary cluster (cluster 1) spanned from 2006 to 2010 with a *RR* of 2.71 (*P*-value < 0.001), which covered 102 communities and centered by Zhangjiang Community (Pudong new area). The spatial distribution of chronic HCV infection formed one primary cluster (cluster 2) and three secondary clusters (cluster 3 to 5) as below. Cluster 2 was located at Zhoupu Community (Pudong new area) with a *RR* of 14.42 (*P*-value < 0.001), spanned from 2017 to 2018. Cluster 3 covered 43 communities and centered by Sitang (Baoshan District), with a *RR* of 1.94(*P*-value < 0.001), spanned from 2016 to 2018. Cluster4 was located at Fenglin Community (Xuhui District) with a *RR* of 6.04 (*P*-value < 0.001), spanned from 2015 to 2017. Cluster 5 was located at Qingpu Community (Qingpu District) with a *RR* of 4.58(*P*-value < 0.001) spanned from 2016 to 2017. Among the total 240 communities, 115 (47.92%) communities were classified as hotspot regions. According to the spatial distributions of acute and chronic HCV infections, 34 communities (accounting for 29.56% of the hotspot communities) were revealed as overlapping areas.

**Table 2:** HCV infection spatiotemporal clusters with high rates identified by SaTScan discrete *Poisson* method, Shanghai, China, 2005–2018

Cluster	1	2	3	4	5
Time period	2006–2010	2017–2018	2016–2018	2015–2017	2016–2017
Annual incidence. obs (/100,000)	1.40	100.98	12.78	42.83	32.59
Annual incidence. exp (/100,000)	0.67	7.17	7.18	7.17	7.18
*RR*	2.71	14.42	1.94	6.04	4.58
*LLR*	236.13	462.88	292.12	148.68	86.19
*P* value	< 0.001	< 0.001	< 0.001	< 0.001	< 0.001
No. communities	102	1	43	1	1

Cluster 1 belongs to primary clusters of acute HCV infection;

Cluster 2 belongs to primary clusters of chronic HCV infection, and cluster 3, 4 and 5 belong to secondary clusters of chronic HCV infection;

Annual incidence. Obs, annual incidence/prevalence of observed cases;

Annual incidence. Exp, annual incidence/prevalence of expected cases;

*RR*, relative risk for the HCV incidence in the cluster compared to the national average incidence at the same time period;

*LLR*, log likelihood ratio;

No. communities, number of communities within cluster

Hot spot analysis of 86 hospitals was furtherly mapped on Fig. 2, and 5 hospitals were identified as cluster effects, including Hospital B (*Z* = 4.371, *P*-value < 0.001), Hospital A (*Z* = 4.143, *P-*value < 0.001), Hospital E (*Z* = 3.957, *P*-value < 0.001), Hospital C (*Z* = 2.790, *P*-value = 0.005) and Hospital D (*Z* = 1.985, *P*-value = 0.047).

### HCV genotype determination and mapping

From 2014 through 2018, a total of 940 blood samples of HCV patients have been collected from 24 sentinel hospitals and examined in the laboratory of SCDC. Of them, 773 samples tested positive for HCV nucleic acid and 739 samples tested positive for anti-HCV antibody. Furtherly, 517 samples were genotyped successfully based on both fragments (n = 395) or one fragment (5’UTR, n = 91; NS5B, n = 26), there were 5 samples with inconsistent genotyping results; thus, the genotypes were determined by 5’UTR, the remaining 256 samples were discarded due to poor quality reads. Four genotypes were identified based on 5’UTR and NS5B sequences, including GT1 (N = 257, 49.71%), GT2 (N = 34, 6.58%), GT3 (N = 165, 31.91%), and GT6 (N = 61, 11.80%). GT1b, GT3a and GT3b accounted for 47.20% (244/517), 16.83% (87/517) and 14.70% (76/517) of HCV infections, respectively. GT6, including 6a, 6b, 6n, 6u, 6v and 6xe, totally accounted for 11.80% (61/517) of HCV infections in Shanghai. Details were shown in Table 3.

**Table 3:** The time distribution of HCV subtypes, Shanghai, China, 2014–2018

Year	No. of samples	No. of determinations	Subtypes					
			1b	3a	3b	6a	1a	Others^a^
2014	71	39	18 (46.15)	8 (20.51)	5 (12.82)	2 (5.13)	2 (5.13)	4 (10.26)
2015	263	151	83 (54.97)	26 (17.22)	7 (4.64)	12 (7.95)	4 (2.65)	19 (12.58)
2016	152	99	38 (38.38)	19 (19.19)	18 (18.18)	4 (4.04)	5 (5.05)	15 (15.15)
2017	193	106	46 (43.40)	13 (12.26)	24 (22.64)	6 (5.66)	0 (0)	17 (16.04)
2018	261	122	59 (48.36)	21 (17.21)	22 (18.03)	2 (1.64)	1 (0.82)	17 (13.93)
Total	940	517	244 (47.20)	87 (16.83)	76 (14.70)	26 (5.03)	12 (2.32)	72 (13.93)

^a^Other subtypes include 1c, 2a, 2c, 2i, 3k, 6b, 6k, 6n, 6u, 6v, 6xe

**Fig. 3:**
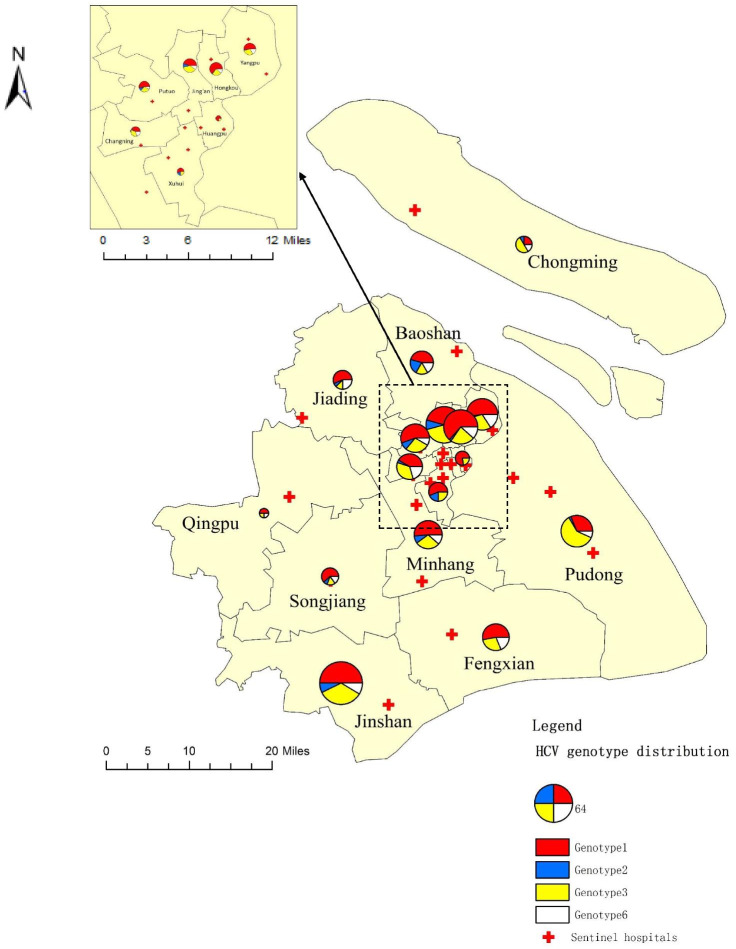
Geographic distribution of HCV genotypes among districts in Shanghai, China, 2014–2018

HCV genotypes distribution differed across the districts from 2014 through 2018 in Shanghai (Fig. 3). GT1 was distributed in all the districts, which varied between 25.00% (3/12) in Chongming District to 77.78% (7/9) in Huangpu District. GT2 and GT6 were mainly detected in urban area with the highest proportion of 20.83% (5/24) in Baoshan district and 20.69% (6/29) in Changning District, respectively. GT3 was mostly in the suburban area, especially in Pudong new area, with a peak of 60.00% (27/45).

## Discussion

This study showed that Shanghai is a low-prevalence city in China for HCV infection. The incidence of acute HCV fluctuated between 2.16 to 0.12 per 100,000 persons with a peak in 2010 then steadily kept down to a pretty low level, the prevalence of chronic HCV was registered from 2013 and showed an upward trend with a peak in 2017. These are related to changing requirement of NIDRIS under certain circumstances, viral hepatitis C was required to report instead of non-A non-B viral hepatitis in 2005 and then classified as acute HCV and chronic HCV in 2013 based on suspected exposure history in the last 6 months or biopsy results of the patients [22, 23], thereafter Screening and management of viral hepatitis C was published in 2014 [24], strengthening the HCV screening in need, all the efforts had improve the surveillance quality of HCV, to better monitor the true prevalence state. The mean age of chronic HCV patients was about 3 years older than acute ones, which indicated spontaneous clearance for the natural history, as well as a few acute HCV cases may not get timely and standard treatment. These all have led to a major understanding of the epidemiological features of hepatitis C in Shanghai.

According to the results of global auto-correlation analysis at community scale, the distributions of both acute and chronic HCV cases in Shanghai were spatially clustering in most years, whereas the value of Moran’s *I* was low, which inferred HCV infection may vary among different communities in Shanghai. Local auto-correlation analysis showed the clusters of acute and chronic HCV cases were relatively scattered and irregular from year to year. To further explore the temporal and spatial factors in hepatitis C distribution, SaTScan was used to detect and five time-spatial clusters for HCV infection appeared, including one primary cluster for acute HCV infection, one primary cluster for chronic HCV infection, and three secondary clusters for chronic HCV infection. Four hospitals were identified to have close relationship with clusters 1 to 4, Hospital B and Hospital A are related to Cluster 1 and 3, respectively, Hospital D is related to cluster 2, and Hospital C, cluster 4. It may partly explain the existence of the clusters. Additionally, we had another interesting finding of an overlap of 34 communities occurred between acute and chronic HCV clusters and the time-span varies from 6 to 12 years, which were consistent with the nature history of HCV infection progression from asymptomatic acute stage to chronic stage with fibrosis and even cirrhosis. It also indicated that some acute patients may not receive effective treatment in the past decade and suggested early detection and active treatment were of great importance.

The analysis of the results of HCV genotype determination revealed geographical difference from other regions of China. GT1b was still the most prevalent HCV genotype in Shanghai, however the proportion (49.71%) is lower than China as a whole, where 1b accounting for 62.78% [25], meanwhile, GT3a and 3b possessed the second and third place instead of 2a. GT6 presented highly diversities, in line with other studies carried out in China [26, 27]. Based on tons of epidemiological researches, the infections of some specific HCV genotypes are closely related to certain transmission routes [28, 29]. Specifically, GT3 and GT6 are mainly transmitted through intravenous drug using, and prevalent in drug users [30], while GT1 and GT2 are inclined to having exposure history of blood donation and invasive procedures before 1993 in China when HCV nucleic acid detection technology had not been introduced widely [7]. According to the research of Lu [31], GT3 infectors tend to progress into liver cirrhosis than other genotypes, which need intensive interventions and focused elimination strategies.

Further analysis of the distribution of HCV genotypes found, Baoshan district met the highest proportion of GT2, and Pudong new area occupied the highest proportion of GT3. Combined with the results of the time-spatial clustering analysis, large part of Baoshan was the cluster of chronic hepatitis C, and most areas of Pudong presented clusters of acute hepatitis C, which implied the differentiation of transmission route distribution among different regions of Shanghai. We inferred, as Baoshan district nearing the Qidong city (Jiangsu, China) with a high HBV infection rate [32], people from Qidong city tend to settle down in Shanghai city at this region, it may increase the vulnerability of residents living in this district. As for Pudong new era, it may due to high proportion of migration population including drug users from other provinces of China like Yunnan and Xinjiang [33]. Thus, initiating tailored prevention strategies if there is in need. It is still of significance to monitor the prevalence of HCV cases with specific genotypes regularly, which can inform the strategies of HCV prevention and treatment effects.

Our study has several limitations. First, cases information was released from NIDRIS, the accuracy of the results was closely connected with the reporting criteria. As mentioned before, the reporting criteria changed separately in 2005 and 2013. Especially in 2013, viral hepatitis C was classified as an acute and chronic case, thus, an overlap between acute and chronic may exist during 2005–2012, and the ratio was hard to estimate. Second, five clusters were detected through the time-spatial clustering analysis, among them, four clusters may be closely related to the existence of famous or special hospitals. The famous hospital effect may lead to overestimation of the value of log-likelihood ratio of the clusters and exaggerate the circumstances of HCV infection among residents in Shanghai. Third, the blood samples in this study were directly collected from the laboratory of the sentinel hospitals and were labeled according to the geographic information of the hospitals, thus we cannot distinguish between residents and non-residents, nor can we map the HCV genotypes/subtypes based on their residential address and further analyze it from the geographical aspect, data collecting process should be strengthened to identify the presence of different hot spots of certain genotypes/subtypes in the future. Another limitation is the determination of HCV genotypes, since genotyping results may differ based on the different fragments of the HCV genome. In our study, we obtained inconsistent genotyping results in 5 samples, in addition, we could not complete both genotyping examinations due to the shortage of blood samples (117/517). It warranted further improvement in the sample collection in the sentinel hospitals.

## Conclusions

In this study, we provided a comprehensive epidemiological overview of HCV and mapped the dynamics of different HCV subtypes distribution, inferred more clusters of asymptomatic HCV-infected individuals according to the spatial and temporal characters of HCV cases for cure and elimination in Shanghai, which can contribute to the elimination strategies for the low-prevalence areas in China.

There exists a time-spatial clustering pattern of HCV infection in Shanghai, despite the low prevalence of HCV infection, it is still needed to push forward the elimination process in Shanghai, as there is a certain amount of HCV infected people waiting to be treated. The time-spatial clustering patterns and the dynamic of HCV genotype distribution together indicated a changing constitution of different transmission routes of HCV infection, thus, a focused strategy may be needed for high-risk population related to genotype 3 infection like drug users, in addition to an enforcement of the existing measures of preventing the iatrogenic and hematogenic transmission of HCV.

In response to the proposal of WHO to eliminate viral hepatitis as a major public health threat by 2030 and the project of viral hepatitis control and prevention in China from 2017 to 2020 [12], Shanghai has prepared to initiate a program of early detection and active treatment of HCV infection. Up to now, hospitals in Shanghai are required to conduct hepatitis testing on each patient before invasive procedures. 117 voluntary counseling testing points at CDC or community health care center can provide free detection and referral services of HBV and HCV. For the next step, HCV rapid testing will be added to the physical examination list such as people over 60 years old.

## Data Availability

The datasets used and/or analysed during the current study are available from the corresponding author on reasonable request.

## Abbreviations

HCV: Hepatitis C virus
HBV: Hepatitis B virus
DAAs: Direct-acting antivirals
GT: Genotype
NIDRIS: National Infectious Disease Reporting Information System
CCDC: Chinese Center for Disease Control and Prevention
SCDC: Shanghai Municipal Center for Disease Control and Prevention
HCC: Hepatocellular carcinoma
AIDS: Acquired Immune Deficiency Syndrome
WHO: World Health Organization
Anti-HCV: Antibody of hepatitis C virus
